# Pro-oxidant Actions of Carotenoids in Triggering Apoptosis of Cancer Cells: A Review of Emerging Evidence

**DOI:** 10.3390/antiox9060532

**Published:** 2020-06-17

**Authors:** Juhyun Shin, Min-Ho Song, Jae-Wook Oh, Young-Soo Keum, Ramesh Kumar Saini

**Affiliations:** 1 Department of Stem Cell and Regenerative Biotechnology, Konkuk University, Seoul 143-701, Korea; junejhs@konkuk.ac.kr (J.S.); ohjw@konkuk.ac.kr (J.-W.O.); 2 Department of Crop Science, Konkuk University, Seoul 143-701, Korea; tdasgtaasf@gmail.com (M.-H.S.); rational@konkuk.ac.kr (Y.-S.K.); 3 Institute of Natural Science and Agriculture, Konkuk University, Seoul 143-701, Korea

**Keywords:** astaxanthin, fucoxanthin, β-carotene, lycopene, bixin, apocarotenoids, p53, anthracyclines

## Abstract

Carotenoids are well known for their potent antioxidant function in the cellular system. However, in cancer cells with an innately high level of intracellular reactive oxygen species (ROS), carotenoids may act as potent pro-oxidant molecules and trigger ROS-mediated apoptosis. In recent years, the pro-oxidant function of several common dietary carotenoids, including astaxanthin, β-carotene, fucoxanthin, and lycopene, has been investigated for their effective killing effects on various cancer cell lines. Besides, when carotenoids are delivered with ROS-inducing cytotoxic drugs (e.g., anthracyclines), they can minimize the adverse effects of these drugs on normal cells by acting as antioxidants without interfering with their cytotoxic effects on cancer cells as pro-oxidants. These dynamic actions of carotenoids can optimize oxidative stress in normal cells while enhancing oxidative stress in cancer cells. This review discusses possible mechanisms of carotenoid-triggered ROS production in cancer cells, the activation of pro-apoptotic signaling by ROS, and apoptotic cell death. Moreover, synergistic actions of carotenoids with ROS-inducing anti-cancer drugs are discussed, and research gaps are suggested.

## 1. Introduction

**Figure 1:**
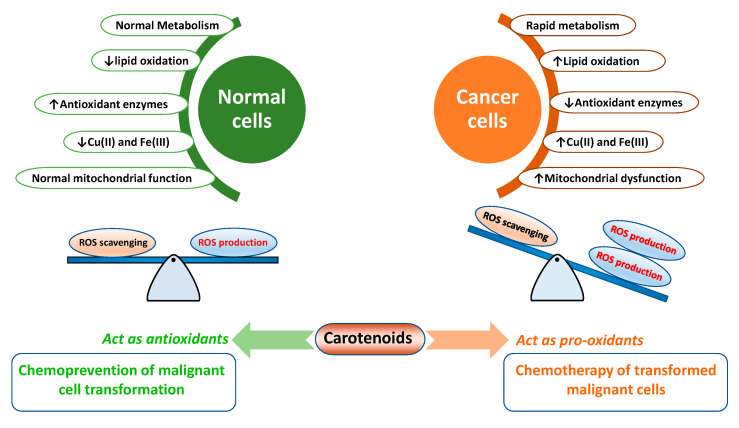
An outline of major differences in cellular metabolism between normal and cancer cells. Under pre-existence conditions of high oxidative stress in cancer cells, carotenoids predominantly function as pro-oxidants, resulting in the ROS-triggered apoptosis of cancer cells.

In the human body, a range of short-lived reactive oxygen species (ROS), including superoxide radical/anion (O_2_^•−^), hydroxyl radical (^•^OH), and hydrogen peroxide H_2_O_2_ are endogenously produced by mitochondria (electron transport chain), NADPH oxidase, peroxisomes, and endoplasmic reticulum (as byproducts during the cellular metabolic process) [1]. ROS at low and regulated levels play an essential role in metabolism, signaling, cellular growth, and development [2]. ROS generation is significantly elevated after exposure to exogenous stresses such as pollutants, ionizing radiation, heavy metals, and xenobiotics (e.g., antiblastic drugs), resulting in substantial damage to DNA and other biomolecules of cells [1]. The imbalance between ROS production and ROS accumulation with the inability of cells to detoxify these harmful substances is termed oxidative stress. It has been well established that malignant cells can innately maintain high intracellular ROS levels compared to normal cells [3,4]. Rapid metabolism, mitochondrial malfunction, increased levels of lipid oxidation, and metal ions (e.g., Cu(II)) [5], lower the levels of antioxidant enzymes (e.g., superoxide dismutase (SOD), glutathione (GSH) peroxidase (GPX), GSH reductase (GR), and catalase). Meanwhile, the impairment of endogenous antioxidants [3] in cancer cells is probably responsible for high intracellular ROS levels. Enhanced levels of oxidative stress in cancer cells can help cancer cells retain their rapid proliferation. However, they are inadequate to cause apoptosis. Thus, further elevating ROS stress using chemotherapeutic drugs (such as paclitaxel, cisplatin, doxorubicin, arsenic trioxide, and etoposide) and phytochemicals, including carotenoids, has been found to be a promising approach to kill cancer cells through cytotoxicity (Figure 1).

Carotenoids are yellow, orange, and red tetraterpenoid pigments universally synthesized by all terrestrial and aquatic photoautotrophs, including plants, algae, some non-photosynthetic bacteria, some insects, and some fungi [6]. Since animals (excluding some species gall midges, pea aphids, and spider mites) are incapable of biosynthesizing carotenoids [7], they need to obtain carotenoid from their diets.

Although more than 40 carotenoids are present in the human diet, only six carotenoids, namely lycopene (bright red pigment of tomatoes), α- and β-carotene (most common precursors of vitamin A), lutein (major carotenoids in green leafy vegetables), zeaxanthin, and β-cryptoxanthin (major carotenoid in citrus fruits), representing >95% of total carotenoids present in the blood [8]. In the body, carotenoids are principally stored in adipose tissues and the liver [9]. Concentrations of carotenoids in the body vary significantly depending on gender, geographical regions, smoking status, and body mass index [10]. In human plasma, lycopene is dominantly found (0.43–1.32 μM; among Europeans), followed by lutein (0.26–0.70 μM), β-carotene (0.21–0.68 μM), β-cryptoxanthin, α-carotene, and zeaxanthin [10].

Vitamin A (retinol, retinal, and retinoic acid) is an essential nutrient for humans, converted mostly from the retinyl palmitate found in animal-derived food. Humans and other herbivores/omnivores can synthesize vitamin A from carotenoid with a non-substituted beta-ionone ring, such as α-carotene, β-carotene, and β-cryptoxanthin dominantly found in fruits, vegetables, and algae [6]. Therefore, for vegetarians, fruits, vegetables, and algae are the major dietary sources of pro-vitamin A carotenoids.

The dietary reference intake (DRI) of carotenoids is not yet established. Currently, the only available carotenoid DRI is for vitamin A, expressed as the retinol activity equivalent (RAE), which considers the varied levels of bioavailability from different sources. The DRI is 300–400 RAE for children, 900 and 700 RAE for males and females, respectively, with a higher intake during pregnancy (770 RAE) and when lactating (1300 RAE) [11]. One RAE is considered equivalent to 1 μg of retinol, 2 μg of β-carotene dissolved in oil, 12 μg of dietary β-carotene, and 24 μg of other dietary pro-vitamins A carotenoids [11]. As no recommendations are established for carotenoid intake, dietary intake of 10–20 mg/d of lutein and 5.7–15 mg/d of lycopene has been suggested for obtaining the health benefits associated with these bioactives [12,13].

Under normal cellular conditions, carotenoids are powerful antioxidants that can help adjust oxidative stress in cells [14,15]. In cancer cells with innately high intracellular ROS levels, carotenoids may act as pro-oxidants and trigger ROS-mediated apoptosis of cancer cells. In recent years, several dietary carotenoids, including astaxanthin, β-carotene, and lycopene have shown pro-oxidant actions that can mediate apoptosis of cancer cells. Besides, when carotenoids are delivered with ROS-inducing cytotoxic drugs, carotenoids can minimize the harmful effects of these cytotoxic drugs to normal cells (an antioxidant mechanism) without interfering with the cytotoxicities of drugs to cancer cells (a pro-oxidant mechanism). This review discusses possible mechanisms involved in carotenoids-triggered ROS production in cancer cells, the activation of pro-apoptotic signaling by ROS, and apoptosis. Moreover, a synergistic action of carotenoids with ROS-inducing anti-cancer drugs is discussed.

## 2. Carotenoids in Biomembrane: Why Different Carotenoids Behave Differently?

Carotenoids are diverse molecules with varying physical, chemical, and biological properties [6]. Among major carotenoids found in the diet, α-carotene, β-carotene, and lycopene are classified as carotenes [16]. A simple hydrocarbon structure and the absence of functional groups make them non-polar. Oxygenated derivatives are known as xanthophylls. In xanthophylls, the oxygen atom can be present in the form of a hydroxyl- (-OH) (e.g., lutein, zeaxanthin, and β-cryptoxanthin), a carbonyl- (=O) (e.g., canthaxanthin), a combination of hydroxyl- and carbonyl- (e.g., astaxanthin), or as esters of hydroxyl-groups (e.g., fucoxanthin) [17]. The presence of these diverse functional groups with *cis*- (-*Z*) and *trans*- (-*E*)-configuration of hydrocarbon backbone influences their polarity and biological functions. In general, the *Z*-isomers of carotenoids possess more polarity, bioavailability, and antioxidant activity than their *E*-counterpart [18,19]. Moreover, carotenoids with varied polarity and functional groups are incorporated differently in the phospholipid-bilayer. Apolar carotenes (e.g., β-carotene and lycopene) are found deep inside the hydrophobic core of the biomembrane [20]. Such incorporation of apolar carotenes alters the packing of phospholipid acyl chains in a fashion that is associated with compelling pro-oxidant actions in lipid peroxidation [20]. On the other hand, polar xanthophylls (e.g., astaxanthin) appear to span within the phospholipid bilayer with their polar functional groups extending toward headgroup regions [20]. Moreover, at different concentrations, carotenoids incorporation in the membrane may alter fluidity, lipid hydrophobicity, and permeability towards other carotenoids, oxygen, free radicals, and toxins [20,21]. For example, from an equimolar mixture of astaxanthin, β-carotene, and lutein (2.5 mM each), astaxanthin has the highest cellular uptake by MCF-7 breast cancer cells, followed by β-carotene and lutein [22]. As expected, the highest cellular uptake of astaxanthin is positively correlated with ROS-production and ROS-induced cytotoxicity in that study [19]. Similarly, in human leukemia HL-60 cells, siphonaxanthin shows 2-fold higher uptake than fucoxanthin (from 10 μM mixture) [20]. Such higher intake is positively correlated with growth inhibitory effects of siphonaxanthin [23]. In addition to the chemical nature of carotenoids, characteristics of cancer cells are also key determinants of cytotoxicity mediated by carotenoids [24]. For instance, estrogen receptor (ER) negative MDA-MB-231 human breast cancer cells are more susceptible to lycopene treatments than ER positive MCF-7 cells [24].

## 3. Carotenoids-Triggered ROS Production in Cancer Cells

There is no uncertainty to proclaim that under optimum concentrations with normal cellular metabolism, carotenoids can act as powerful antioxidants. Several in vitro [25] and human clinical trials have witnessed the protective effects of carotenoids by regulating oxidative stress. In a randomized placebo-controlled clinical trial carried out on 60 volunteers with metabolic syndrome, after treating them with 30 mg/day dose of crocin for 8 weeks, there was a significant decrease (11.7%, *p* = 0.014) in serum pro-oxidant–antioxidant balance compared to the control group [26]. Similarly, in a randomized, double-blind, placebo-controlled study on free-living healthy adults, 2–8 mg of astaxanthin/d supplementation for 8 weeks (plasma concentrations of 0.13 μM in 8 mg group after 4 weeks) enhanced immune response and decreased DNA damage biomarker (plasma 8-OHdG) and acute-phase protein (C-reactive protein) levels [27]. Potent antioxidant properties of carotenoids can protect against chronic diseases, including cancer, cardiovascular diseases, and neurodegenerative disorders [28,29].

Carotenoids are recognized mostly for their antioxidant and cytoprotective properties, however, at high concentrations and under unusual conditions such as unbalanced and high intracellular oxidative stress (common in cancer cells), high oxygen tension (lungs of smokers), low levels of endogenous enzymes and antioxidants, and higher levels of reactive metal ions (e.g., Fe (III) and Cu(II)), carotenoids can function as pro-oxidants [9,14,30,31]. In vitro investigations have suggested that lycopene and β-carotene are powerful antioxidants at low oxygen partial pressure (pO2 < 150 Torr; 200 mbar) [31,32]. However, they are autoxidized easily to exhibit potent pro-oxidant actions at high pO2 [31,32].

Compared to normal cells, malignant cells produce and maintain high intracellular ROS levels [3] due to their lower levels of antioxidant enzymes (e.g., SOD, catalase, GPX, and GR) and endogenous antioxidants (e.g., tocopherols and ascorbate) [3], thereby hampering normal detoxification of radical species. Moreover, compared to normal cells, cancer cells have higher concentrations of metal ions such as Cu(II) and Fe(III) that are probably responsible for Fe(III)–Fe(II) and Cu(II)–Cu(I) reduction and generation of ^•^OH and OH^−^ from H_2_O_2_ (Fenton reaction) [5].

**Figure 2:**
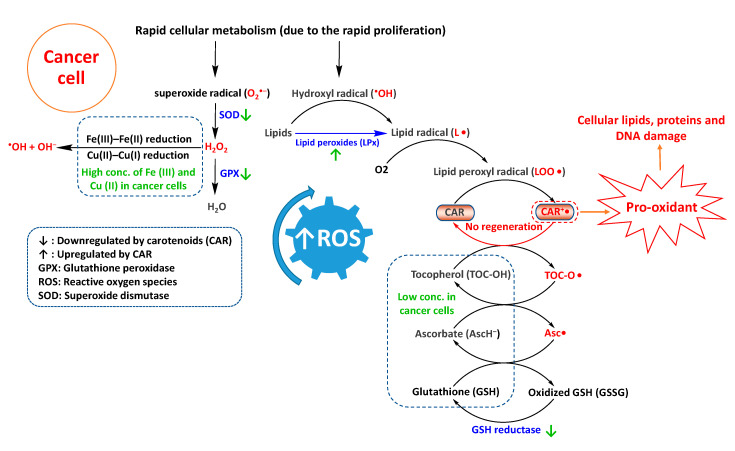
Possible routes of carotenoids-triggered reactive oxygen species (ROS) production in cancer cells.

Under normal physiological conditions, carotenoids can detoxify ROS by several mechanisms, including electron transfer, allylic hydrogen atom abstraction, and radical addition [9]. For instance, upon interaction with lipid peroxyl radical (LOO^•^), the carotenoid can transfer an electron and transform to carotenoid radical cation (CAR^•+^) [9]. For endogenous antioxidants such as ascorbate (redox potential (E°) of 282 mV, Asc^•^, H^+^/AscH^−^) and tocopherol (E° of 500 mV, TOC-O^•^/TOC-OH) at normal cellular concentrations, radical cation CAR^•+^ (E° of 980–1060 mV) is regenerated to CAR [33,34]. Tocopherols and ascorbate are redox partners of carotenoids. Emerging evidence has suggested that carotenoids perform the best as antioxidants when they have appropriate and balanced concentrations with these redox partners [35]. However, in cancer cells with low concentrations of endogenous antioxidant enzymes, regeneration of CAR^•+^ is probably hindered. In turn, CAR^•+^ can enhance ROS levels by catalyzing and propagating the radical chain reactions (a pro-oxidant action) [35], resulting in damage to cellular lipids, proteins, and DNA (Figure 2). Moreover, non-regenerated pro-oxidant CAR^•+^ may autoxidize into apo-carotenals, apo-carotenols, and epoxides that can further enhance redox levels [36].

In addition to carotenoids, several other well-known antioxidative phytochemicals, including polyphenols [37], ascorbate [38], and tocopherols have shown pro-oxidant activities under certain physiological and biochemical conditions [39]. By employing a variety of cancer cell lines and mice bearing xenografts models, it has been shown that ascorbate at pharmacologic concentrations is a pro-oxidant that can generate ascorbate radical- and H_2_O_2_-dependent cytotoxicity toward most of the investigated cancer cells and inhibit tumor progression in xenograft mice models without displaying toxicity to normal tissues or cells [38]. It has been shown that ascorbate is oxidized to form ascorbate radical (AA^•^) by putative metalloprotein catalyst(s) (10–30 kDa) [35]. Ascorbate radical (AA^•^) can donate an electron to O^−^_2_ and form tumoricidal effector H_2_O_2_ [35]. It has been demonstrated that the specific cytotoxicity of ascorbate to cancer cells is H_2_O_2_-dependent because the addition of catalase can decrease the cytotoxicity of ascorbate to sensitive cancer cells [35].

Based on the relative oxidation rates measured by peroxide formation upon pure methyl linoleate addition, major carotenoids can be classified in three categories: (I) carotenoids with little antioxidant properties (e.g., ζ-carotene and phytoene), (II) good antioxidants with condition-dependent prooxidant activities (β-carotene and lycopene), and (III) powerful antioxidants without pro-oxidant activities that can quench ground state as well as excited state radicals (e.g., astaxanthin and canthaxanthin) [32]. However, a recent study has shown that astaxanthin at 20 µM has a substantial pro-oxidant effect (53.3% increase in ROS, compared to 17.3% increase in control) on MCF-7 cells and this effect is synergistically enhanced when cells are treated with a mixture of astaxanthin, β-carotene, and lutein (68.1% increase in ROS) [22]. These observations suggest that while astaxanthin is a well-known antioxidant that protects cells from oxidative injury [27], it may also induce oxidative stress in cancer cells.

Among carotenoids, β-carotene is the most widely investigated one for its pro-oxidant actions in several in vitro, in vivo, and human studies [9,40,41]. Under low- to -moderate levels of oxidative stress, β-carotene functions as a potent chain-breaking antioxidant towards lipid ROO^•^ by scavenging ^1^O_2_. [41]. However, under high oxygen tension conditions (e.g., in the lungs of smokers), β-carotene shows tumor-promoting effects. This has prompted several human clinical trials, leading to suggestions against its supplementation to smokers [41,42]. Moreover, in lung epithelial BEAS-2B cells, β-carotene can inhibit the activity of glutathione-*S*-transferase π (GSTπ), an enzyme that provides protection form benzo[a]pyrenediolepoxide, a tobacco-derived carcinogen [42]. Under high concentrations (e.g., β-carotene breakdown products concentrations of 0.5–20 µM in rat liver mitochondria), high oxygen tension, and unbalanced high intracellular oxidative stress conditions, the pro-oxidant properties of β-carotene dominate its antioxidant properties [9,40,41].

## 4. Enhanced ROS as a Cancer Cure: Mechanism of Carotenoid Triggered Apoptosis

**Figure 3:**
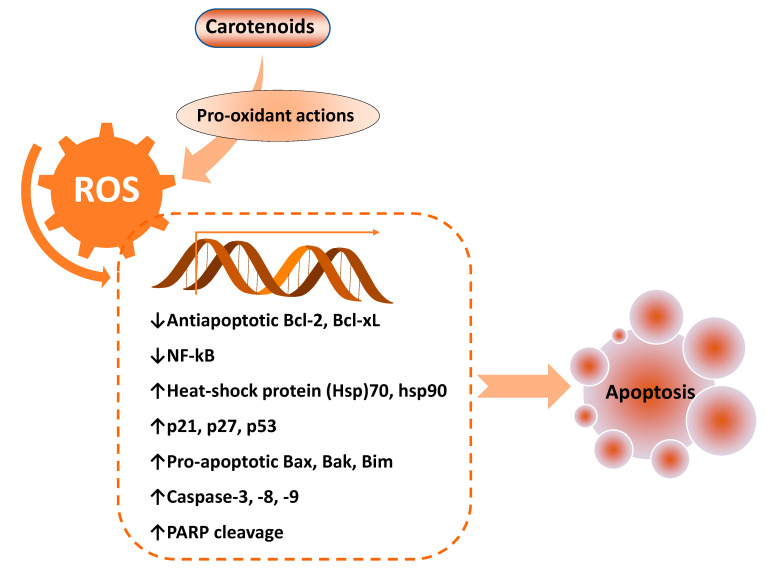
Key pro-apoptotic and anti-apoptotic factors regulated by enhanced reactive oxygen species (ROS) levels, mediated by pro-oxidant effects of carotenoids. Bcl-2: B-cell lymphoma 2; Bak: Bcl-2 homologous antagonist/killer; Bax: Bcl-2-associated X protein; Bcl-xL: Bcl-extra-large; Bim: Bcl-2-like protein 11; NF-kB: Nuclear factor-κB; PARP: poly (ADP-ribose) polymerase.

ROS has both pro- and anti-tumorigenic roles in cancer cells. At low concentrations, an increase in cellular ROS can trigger pro-tumorigenic signaling, enhancing cell proliferation and survival. However, at higher concentrations, an increase in ROS can boost anti-tumorigenic signaling and trigger oxidative stress-induced apoptosis of cancer cells [43]. ROS generation has been investigated as a key mechanism that triggers apoptosis of cancer cells upon treatment with several carotenoids, including Bixin [44], β-cryptoxanthin [45], lutein [15], lycopene [14,36], astaxanthin [22], and fucoxanthin (8–30 µM) [46] (Table 1). It has been shown that enhanced activities of pro-apoptotic proteins p21, p27, p53, and B-cell lymphoma 2 (Bcl-2)-associated X protein (Bax) with decreased activities of anti-apoptotic Bcl-2 and Bcl-extra-large (Bcl-xL) proteins accomplish this ROS-triggered apoptosis (Figure 3).

ROS generation by autoxidation or chemically (KMnO_4_) oxidized lycopene products at 1–50 μM (e.g., Apo-1,6’-carotendial and Apo-5,6’-carotendial) has been proposed as an intermediary stage toward apoptosis in several human cancer cell lines, including PC-3 (prostate), HeLa (cervical), and MCF-7 (breast) [14]. Treatment with Apo-1,6’-carotendial and Apo-5,6’-carotendial not only can lead to a ROS increase, but it can also mediate nuclear condensations, morphological changes, increased malondialdehyde (MDA) levels, and depleted GSH levels in cancel cell lines [14]. Chemically oxidized products are more efficient than autoxidized products in ROS generation, subsequently triggering more apoptosis of cancer cells [11]. In another study, after autoxidized or KMnO_4_ oxidized lycopene products are separated by silica column chromatography, their apoptotic potentials against breast cancer cell line MCF-7 have been investigated [36]. Among a total of three fractions, fraction II that is enriched in the apo-8,6′-carotendial (50–100 µM) shows the highest apoptotic effects (IC50 value of 64.5 μM) probably due to its high cellular uptake because of its appropriate structure, polarity, and solubility, thereby showing enhanced ROS generation [33]. Interestingly, treatment with fraction II at a concentration of 50 µM resulted in significantly higher levels of ROS compared to that at 100 µM, while 100 µM treatment resulted in a nearly three-fold increase of apoptotic cells compared to 50 µM treatment [33]. Treatment with fraction II at 50 µM increased the ROS level by 10.1% and apoptosis by nearly three-fold compared to the lycopene treatment [33]. These observations suggest that fraction II has an apoptotic effect on MCF-7 cells in ROS-dependent (at 50 µM concentration) and ROS-independent (at higher concentrations of 100 µM) manners.

A previous study has shown that co-treatment of astaxanthin with equimolar β-carotene and lutein (2.5 µM each) can synergistically enhance cellular uptake, cytotoxicity, and oxidative stress compared to treatment with individual carotenoids at a similar concentration [22]. The co-treatment can synergistically induce apoptosis through a ROS increase (68.1%, compared to 17.3% in control), MDA increase (≈2.5 fold higher than control), GSH decrease (≈2 fold lower than control), enhanced expression of p53 and Bax, and down-regulation of Bcl-2 proteins [22]. Interestingly, after individual treatment or equimolar treatment with astaxanthin, β-carotene, and lutein, astaxanthin showed the highest cellular uptake, followed by β-carotene and lutein [19]. Higher uptake presumably will lead to higher apoptotic activity [22].

ROS generation has also been demonstrated to be a crucial intermediary step in fucoxanthin triggered apoptosis of a leukemia cell line [46]. After HL-60 cells were treated with 8–30 µM fucoxanthin, when both intracellular H_2_O_2_ and superoxide ions were recorded using fluorescence-activated cell sorting (FACS) for 2’7’-dichlorodihydrofluorescein diacetate (DCFH_2_-DA) probe and dihydroethidium (DE), it was found that intracellular ROS in HL-60 cells was increased by fucoxanthin in a dose-dependent manner. Meanwhile, no significant changes were detected in the human colon cancer HCT-15 or human hepatic carcinoma HepG2 cell line [46]. Enhanced ROS levels in HL-60 were found to be concordant with enhanced expression levels of cleaved caspases-3 and -7, and poly (ADP-ribose) polymerase (PARP) and a substantial decrease in the Bcl-xL protein level. In further validation experiments, N-acetylcysteine (NAC) treatment reversed fucoxanthin-triggered upregulation of caspase-3, caspase-7, PARP cleavage, and apoptosis. NAC functions as a rapid-acting antioxidant by triggering the intracellular production of oxidant scavengers, such as hydrogen sulfide and sulfane sulfur [51]. In contrast, in a previous experiment [52], apoptosis induced by 10 µM fucoxanthin in HL-60 cells was not associated with an altered expression level of ROS, Bcl-2, Bcl-xL, or Bax protein. In that study [45], fucoxanthin caused the substantial loss of mitochondrial membrane potential (ΔΨm) at an early stage of apoptosis, followed by the enhanced cleavages of caspase-3 and PARP proteins. Intracellular H_2_O_2_ recorded by measuring with DCFH_2_-DA probe showed no significant change. These discrepancies were probably caused by whether an effective concentration of fucoxanthin was applied to HL-60 cells.

In our recent study using HeLa cells, treatment with lutein (1 to 10 µM) also enhanced ROS [15]. The treatment resulted in considerable damage to nuclear DNA, downregulation of anti-apoptotic Bcl-2 mRNA expression, and upregulation of pro-apoptotic Bax mRNA [15]. In that study, lutein triggered an increase in the ratio of Bax and Bcl-2, which subsequently caused a loss of ΔΨm, resulting in caspase-3 mediated apoptosis. Similar results have been achieved in HeLa cells after treatment with 1.0 and 10 µM β-cryptoxanthin [45]. In that study, treatment with β-cryptoxanthin triggered an increase in the ROS level, resulting in considerable damage to nuclear DNA, which subsequently caused a significant loss of ΔΨm, upregulation of cleaved caspase-3 proteins, and apoptosis.

## 5. p53: A Key Mediator of ROS-Induced Apoptosis

Tumor protein p53, also known as tumor suppressor p53, is a redox-active transcription factor that coordinates key cellular responses (e.g., cell cycle arrest and apoptosis) to genotoxic stresses [53]. As the most commonly mutated gene in human cancer, p53 is a crucial protein related to tumor suppression. Mutations of p53 can lead to the loss of tumor suppressor activity or gain of function (GOF) that contributes to pro-survival and enhanced proliferation of malignant tumors [54].

**Figure 4:**
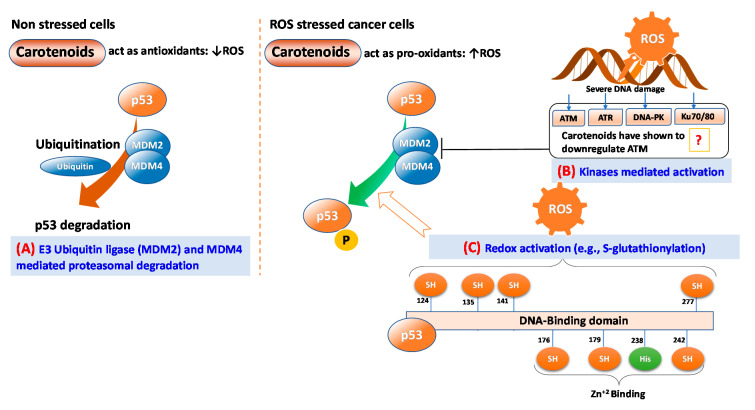
Major routes of p53 activation triggered by carotenoids-mediated reactive oxygen species (ROS)-induced. (**A**) E3 ubiquitin ligases MDM2 and MDM4 mediated proteasomal degradation in non-stress cells; (**B**) Kinases-mediated activation in cancer cells, and (**C**) Direct redox activation by *S*-glutathionylation of redox-sensitive cysteine residues at 124, 135, 141, and 277 of DNA-binding domain. ATM: ataxia telangiectasia mutated; ATR: ataxia telangiectasia and Rad3 related (a serine/threonine-protein kinase); DNA-PK: DNA-dependent protein kinase.

ROS can function as both an upstream signal that activates p53 and as a downstream factor that facilitates apoptosis [53]. In non-stressed cells, proteasomal degradation by mouse double minute 2 homolog (MDM2) also known as E3 ubiquitin ligases and MDM4 can help maintain low levels of p53 [55]. However, in stressed cells such as cells having ROS mediated DNA damage, p53 is stabilized and activated by post-translational modifications, including multi-site phosphorylation, acetylation, and methylation [53]. DNA damage response (DDR) mediates the activation of several kinases, including ataxia telangiectasia and Rad3 related (ATR; a serine/threonine-protein kinase), ataxia telangiectasia mutated (ATM), and DNA-dependent protein kinase (DNA-PK) [56] that activate p53 in coordinated responses [57]. Remarkably, p53 itself is a redox-active protein due to the presence of redox-sensitive cysteine residues at 124, 135, 141, and 277 of the DNA-binding domain [53] (Figure 4). Kinase activated p53 can bind to specific DNA sequences and regulate several potential tumor-suppressive genes related to DNA repair, cell cycle arrest, cell senescence, cell death, and metabolic adaptation (Figure 4).

ATM, ATR, and DNA-PK are members of the phosphoinositide 3 kinase-related protein kinases (PI3KKs) family, which also includes the mammalian target of rapamycin (mTOR) [58]. β-carotene can induce the apoptosis of gastric adenocarcinoma (AGS) cells by increasing p53 and decreasing anti-apoptotic Bcl-2 as well as nuclear ATM [59]. Similarly, lycopene can inhibit the growth of triple (HER2/ER/PR)-negative MDA-MB-468 cells through the upregulation of p21, enhanced cleavage of PARP, and reduced phosphorylation of Protein kinase B (PKB, also known as Akt) and its downstream target mTOR [60]. Moreover, β-carotene can inhibit the growth of MCF-7 cells by increasing the ROS and Akt levels, inhibiting extracellular signal-regulated kinases (ERK)1/2, and activating p53 [61]. In another study, treatment with 20 μM fucoxanthin resulted in ROS-mediated oxidative DNA damage, time-dependent activation of mitogen-activated protein kinases (MAPKs), inhibition of phosphoinositide 3-kinases (PI3K)−AKT, and apoptosis of human glioblastoma cell line U251 [47]. ROS inhibition by antioxidant glutathione (GSH) can inhibit these effects, demonstrating that fucoxanthin-induced oxidation can result in apoptosis [47]. In contrast, in the gastric adenocarcinoma cell line AGS, β-carotene can trigger apoptosis by increasing p53 and Bcl-2, although the oxidative damage marker shows a decrease [59], suggesting that separate routes of p53 activation and apoptosis exist in different carotenoids and cell lines. For example, in the liver cancer cell line HepG2, treatment with β-carotene at 1 to 5 μM inhibits growth signals (such as NF-kB, pAkt, and pERK) and redox signals (such as nuclear factor erythroid 2–related factor 2 (Nrf-2), SOD-2, and heme oxygenase-1 (HO-1)) but triggers apoptotic signals such as cleaved PARP, Bax, and caspase [48].

**Figure 5:**
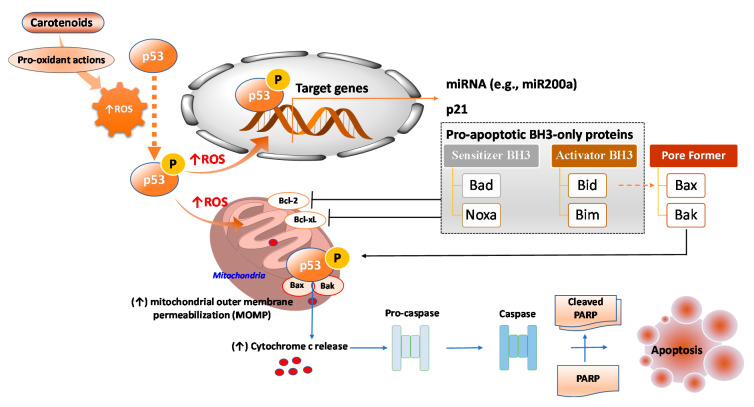
Mechanisms of the carotenoids-mediated p53-induced apoptosis of cancer cells. Bcl-2: B-cell lymphoma 2; Bad: Bcl-2-associated agonist of cell death; Bak: Bcl-2 homologous antagonist/killer; Bax: Bcl-2-associated X protein; Bcl-xL: Bcl-extra-large; Bid: BH3 interacting-domain death agonist; Bim: Bcl-2-like protein 11; Noxa: Latin for *damage*, also known as phorbol-12-myristate-13-acetate-induced protein PMAIP1; PARP: poly (ADP-ribose) polymerase.

Upon activation, p53 facilitates pro-apoptotic responses by transcriptionally upregulating key target genes such as p21, which known to be involved in cell cycle arrest and pro-apoptotic BH3-only Bcl-2 family proteins including the Bcl-2-associated agonist of cell death (Bad), Noxa (Latin for damage, also known as phorbol-12-myristate-13-acetate-induced protein PMAIP1; sensitizer of BH3), BH3 interacting-domain death agonist (Bid), and Bcl-2-like protein 11 (Bim; activator of BH3) [62] (Figure 5). BH3-only sensitizer BH3 protein can bind to anti-apoptotic proteins such as Bcl-2 and Bcl-xL and inactivate them. Such interaction competes with the binding of activator BH3-only proteins and pore-formers protein such as Bax and Bak, leading to their displacement from anti-apoptotic proteins [62]. These interactions lead to increased mitochondrial outer membrane permeabilization (MOMP) (loss of ΔΨm), resulting in the efflux of apoptogenic factors (e.g., cytochrome c) from mitochondria (Figure 5). p53 is not only a key tumor anti-tumorigenic transcription factor; it also mediates apoptosis by transcription-independent mechanisms. For instance, in mitochondria, p53 can directly bind to pore-formers and increase MOMP, thereby triggering apoptogenic factors efflux from mitochondria (Figure 5).

Several recent observations have shown that p53 knockout mice, lacking p21, p53 upregulated modulator of apoptosis (Puma), and Noxa (Latin for damage, also known as phorbol-12-myristate-13-acetate-induced protein PMAIP1; sensitizer of BH3) are not susceptible to tumor development, suggesting that p53 not only can suppress a tumor but also can control numerous cellular processes that substantially contribute to the suppression of cancer cell proliferation [55]. These cellular processes controlled by p53 may include cholesterol biosynthesis, fatty acids β-oxidation, ferroptosis (regulated cell death by the enhanced accumulation of iron-dependent lipid peroxides), and maintenance of genome stability [55].

Carotenoids-mediated increases in levels of oxidative stress can decrease levels of DNA binding and repair proteins (e.g., Ku70/80), resulting in enhanced caspase activation and apoptosis of cancer cells. It has been shown that β-carotene can induce apoptosis of AGS cells by elevating ROS, activating caspase-3, and decreasing the level and DNA binding activity of Ku70/80 proteins [63]. In that study, levels of Ku proteins and DNA binding activities decreased, and apoptotic potential was inhibited after treatment with ROS inhibitor NAC and caspase-3 inhibitor z-Asp(OMe)-Glu(OMe)-Val-Asp(OMe)-CH2F (z-DEVED-fmk), suggesting that reduced levels of Ku proteins are downstream events in caspase-3-mediated apoptosis induced by ROS.

## 6. Carotenoids Act Synergistically with Ros-Inducing Anticancer Drugs

ROS-induced cytotoxic cell killing anti-cancer drugs, particularly anthracyclines (doxorubicin, cisplatin, taxol, and paclitaxel), are widely utilized in the treatment of various cancers [64]. This process is primarily mediated through intracellular H_2_O_2_ elevation, DNA intercalation, and inhibition of topoisomerase II, resulting in the arrest of the DNA repair process, which leads to apoptosis [64]. However, anthracyclines are known to induce severe cardiomyopathy and congestive cardiac failure [64]. Several carotenoids can enhance the toxicity of ROS-inducing anti-cancer drugs. For instance, a study has shown that addition of lutein and β-carotene to ROS-inducing doxorubicin at a low dose of 0.2–3.2 μM is effective against breast cancer cell lines MCF-7 and MDA-MB-231 without influencing the redox status (e.g., lipid peroxides (LPx), ROS, and lactate dehydrogenase levels) in normal breast epithelial MCF 10A cells [49]. In that study, the combined treatment with carotenoids and doxorubicin enhanced apoptosis of cancer cells by depleting GSH, increasing levels of LPx, and ROS, causing mitochondrial dysfunction, activating caspases-3, -8, and -9, upregulating p21, p27, p53 and Bax, and down-regulating Bcl-2.

A recent study has shown that bixin, an apocarotenoid isolated from *Bixa Orellana* L, can elevate ROS levels in human melanoma A2058 cells [44]. Moreover, A2058 has an oncogenic mutation of the valine-to-glutamic acid substitution at position 600 (V600E) of v-raf murine sarcoma viral oncogene homolog B1 (BRAF) that makes it resistant to anti-cancer drug dacarbazine. In this study, treatment with bixin + dacarbazine caused substantially higher toxicity (IC_50_ of 31.85 µM) compared to the individual treatments (IC50 of 40.53 µM for bixin and ≫100 µM for dacarbazine), demonstrating the potential of using this carotenoid as a supplement to anti-cancer drugs for treating cancers [44]. Another study has investigated the effects of several carotenoids, including lutein, chemotherapeutic drugs, and their combinations against MCF-7 and breast cancer cell line MDA-MB-468 harboring a point mutation in p53 [50]. Lutein-mediated growth inhibition of these cells is modulated mainly by enhanced ROS production, similarly to ROS levels increased by chemotherapeutic drugs such as taxane, paclitaxel, and docetaxel. As expected, NAC can attenuate these effects. Among combinations of lutein with various drugs, lutein combined with taxane is more efficient in terms of increasing cytotoxicity. Interestingly, treatment with lutein did not affect the total p53 protein levels in MDA-MB-468 cells, but significantly increased the levels of active phosphorylated p53 and cellular heat shock protein 60 (HSP60) [50]. Phosphorylation of mutant p53 can hinder its function as a negative regulator of any remaining wild-type p53 [65]. Thus, the observation that lutein induces mutant p53 phosphorylation in MDA-MB-468 cells shows that lutein might have potential as a therapeutic drug for cancer with p53 mutation.

Finally, the supplementation of carotenoids as preventive antioxidants can attenuate the adverse side effects of anti-cancer drugs on normal cells. For example, a study has shown that supplementation of pequi oil, a carotenoid-rich oil extracted from pequi (*Caryocar brasiliense*), to ohrlich-solid-tumor-bearing mice can attenuate the adverse side effects of doxorubicin on normal cells. Moreover, pequi oil delivered before tumor inoculation or in parallel with doxorubicin was found to be most effective in increasing lymphocyte-dependent immunity and containing tumor growth [66].

## 7. Conclusions

Carotenoids-triggered DNA damage and ROS generation have been widely investigated. They can activate several tumor-suppressing proteins, including p53. However, how carotenoids-triggered ROS activates p53 is not precisely known yet. ROS are implicated in the phosphorylation of p53 that is mediated by protein kinases such as ATM, ERK, and p38α MAPK [53]. However, in vitro studies have suggested that carotenoids-triggered ROS downregulate these kinases while upregulating p53 expression [61]. Thus, another mechanism, probably carotenoids-mediated redox modification mechanism, might induce conformation changes and p53 activation. Future researches should address the effects of carotenoids-mediated redox modification on p53 activation that lead to the enhanced transcription of pro-apoptotic factors. Such information will add substantial information to understand how p53 is activated under the influence of carotenoids-triggered ROS and DNA damage.

## Figures and Tables

**Table 1:** Summary of in vitro mechanistic studies of carotenoids-triggered apoptosis of cancer cells involving pro-oxidant actions of carotenoids.

Experimental System	Carotenoid	Major Outcome	Conclusion	References
U251-human-glioma-cell	Fucoxanthin (20 μM)	↑ROS, ↑DNA damage, ↑MAPKs, ↑Ser428-ATR, Ser1981-ATM, Ser15-p53, and Ser139-histone ↑Thr183-JNK, Thr180-p38, ↓Ser-AKT, ↓PI3K−AKT	Fucoxanthin-induced oxidation of thiol-containing intracellular antioxidant (e.g., GSH and thioredoxin) or blocked the activities of antioxidant enzymes	[47]
Human hepatocellular carcinoma (HepG2) cells	β-carotene (1–5 μM)	↑MOMP, ↓Bcl-2, PARP, and NF-kB, ↓Akt and ERK1/2, ↓SOD-2 and HO-1, Nrf-2, ↑Bax and cleaved PARP, ( 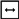 ) ROS	β-carotene suppresses the growth of HepG2 cells by activating the intrinsic apoptotic pathway modulated by intracellular antioxidant status (independent of ROS induction)	[48]
Human melanoma A2058 cells	Bixin (50–100) µM, IC50 of 31.85 µM with bixin + dacarbazine	↑ROS and MDA (a lipid peroxidation marker)	Bixin sensitizes A2058 cells to dacarbazine-induced cytotoxicity through ROS elevations	[44]
HeLa cells	Lutein (1 to 10 µM), IC50 of 7.9 and 3.7 µM after 24 and 48 h of treatments	↑ROS (three times higher ROS in cell treated with 10 µM, compared to control), ↑Bax, p53, caspase-3 mRNA, ↑nuclear DNA damage (97% TUNEL-positive cells in cell treated with 10 µM), ↓Bcl-2	Lutein triggers a ROS-mediated intrinsic apoptotic pathway in HeLa cells	[15]
HeLa cells	β-Cryptoxanthin (1 to 10 µM), IC50 of 4.5 and 3.7 µM after 24 and 48h of treatments	↑ROS, ↑caspase-3, -7, and -9, p53, and Bax mRNA, ↑cleaved caspase-3, ↓Bcl-2 mRNA, ↑MOMP, ↑nuclear DNA damage (52% TUNEL-positive cells in cell treated with 1 µM)	β-Cryptoxanthin triggers ROS-mediated intrinsic apoptotic pathway in HeLa cells	[45]
Breast-cancer MCF-7 cell lines	Autoxidation or KMnO4 oxidized products of lycopene, mainly apo-8,6′-carotendial in fraction II of silica column (IC50 value of 64.5 μM)	↑ROS, ↑mitochondrial dysfunction	ROS-dependent (at 50 µM concentration) and ROS-independent (at higher concentrations of 100 µM) apoptosis of MCF-7 cells	[36]
Estrogen receptor (ER)-positive MCF-7 and ER-negative MDA-MB-231 cells	Doxorubicin (0.2–3.2 µM) + carotenoids (lutein, astaxanthin, β-carotene, and fucoxanthin; 2–10 µM)	↓GSH (29.5–48.8%), ↑LPx, ↑ROS (43.2–65%), ↑mitochondrial dysfunction, ↓Bcl-2, ↑Bax, p53, caspase -3, -8, and -9), p21, and, p27, Cytotoxicity to MCF-7 cells: β-carotene > lutein > fucoxanthin > astxanthin	Carotenoids deliver the synergetic effects towards the cytotoxic killing of cancer cells by ROS-inducing anti-cancer drugs without influencing the redox status and proliferation of normal breast epithelial MCF 10A cells	[49]
Human prostate (PC-3), cervical (HeLa), and breast adenocarcinoma (MCF-7) cells	Autoxidation or chemically (induced by KMnO4) oxidized products (e.g., Apo-1,6’-carotendial and Apo-5,6’-carotendial) of lycopene (1–50 μM)	↑DNA condensations, ↑ROS, ↑MDA, ↓GSH	Chemically oxidized products are more efficient than autoxidation products in ROS generation and enhanced apoptosis of cancer cells	[14]
HL-60	Fucoxanthin (8–30 µM)	↑ROS (fluorescence intensity from 99 (vehicle-treated cells) to 132 (correspondent to superoxide anion, O_2_^•−^), ↑cleaved caspases-3 and -7, PARP, ↓Bcl-xL proteins	ROS-induced enhanced cleavage of caspases-3 and -7, and PARP	[46]
MCF-7	Astaxanthin co-treatment with β-carotene and lutein (equimolar 5 µM each)	↑ROS, MDA (≈2.5 fold higher), ↓GSH (≈2 fold lower), ↑p53 and Bax proteins, ↓Bcl-2 proteins	Astaxanthin works synergistically with β-carotene and lutein to trigger ROS production and the apoptosis of MCF-7 cells	[22]
MCF-7 and triple-negative MDA-MB-468 breast cancer cells	Lutein (2 µM) + chemotherapeutic drugs (e.g., taxanes, paclitaxel and docetaxel; 0.5 µM).	↑ROS (1.9-fold in cells treated with 2 µM lutein compared to control), 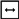 p53 protein, ↑phosphorylated p53 (at serine residues Ser15, Ser46, and Ser392), ↑cellular HSP60	Lutein inhibits the proliferation of breast cancer cells through ROS-mediated activation of p53 protein	[50]

Upregulation and downregulation are symbolized by upward (↑) and downward (↓) arrows, respectively. Similarly, no changes are represented by the 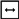 arrow. Akt: Protein kinase B (PKB); ATM: ataxia telangiectasia mutated; ATR: ataxia telangiectasia and Rad3 related (a serine/threonine-protein kinase); Bax: Bcl-2-associated X protein; Bcl-2: B-cell lymphoma 2; Bcl-xL: Bcl-extra-large; DNA-PK: DNA-dependent protein kinases; ERK: signal-regulated kinases; GSH: Reduced glutathione; HO-1: Heme oxygenase-1; HSP: Heat shock protein; JNK: c-Jun N-terminal kinases; LPx: Lipid peroxides; MAPKs mitogen-activated protein kinases; MDA: Malondialdehyde; MOMP: Mitochondrial outer membrane permeabilization; NF-kB: Nuclear factor-κB; Nrf-2: Nuclear factor erythroid 2–related factor 2; PARP: Poly (ADP-ribose) polymerase; PI3K: Phosphoinositide 3-kinases; SOD: Superoxide dismutase.
